# CD4^+^T and CD8^+^T cells profile in lung inflammation and fibrosis: targets and potential therapeutic drugs

**DOI:** 10.3389/fimmu.2025.1562892

**Published:** 2025-05-09

**Authors:** Xiaobo Sun, Xinwen Zhang, Yuhan He, Xueting Du, Qian Cai, Zhihong Liu

**Affiliations:** ^1^ Key Laboratory of Environmental Factors and Chronic Disease Control, Ningxia Medical University, Yinchuan, China; ^2^ Pathogenic Microbiology Laboratory, Yinchuan Center for Disease Control and Prevention, Yinchuan, China

**Keywords:** CD4 ^+^ T cells, CD8 ^+^ T cells, inflammation, pulmonary fibrosis, drug and target

## Abstract

Pulmonary fibrosis is an interstitial lung disease characterized by chronic progressive fibrosis. It is associated with fibrocyte proliferation and collagen deposition, leading to severe, irreversible lung function decline. Despite extensive research, the diagnosis and treatment of pulmonary fibrosis are complicated and have no effective treatment. During the formation of pulmonary fibrosis, immune dysregulation by inflammatory cell infiltration is the key driver of pulmonary fibrosis. Recently, single-cell sequencing analysis of silicosis mice showed that various cells in the alveolar immune microenvironment are involved in forming pulmonary fibrosis, such as macrophages, fibroblasts, epithelial cells, etc. Among them, T cell subpopulations in silicosis mice were significantly activated, indicating that T lymphocyte subsets play an essential role in the process of pulmonary fibrosis. More and more pulmonary clinical studies show that T lymphocytes in the lung immune microenvironment play an important and multifaceted role. This article summarizes the role of CD4^+^T cells and CD8^+^T cells in pulmonary fibrosis. This article provides some new insight into the potential therapy target that can delay the process of pulmonary fibrosis by regulating the proportions of different subpopulations of T lymphocytes and some related signaling pathways.

## 1 Introduction

The pathological process of pulmonary fibrosis is mainly characterized by respiratory impairment due to chronic inflammation and impaired lung tissue repair. Lung tissue repair involves dynamic interactions between stem cells and the immune microenvironment, and immune cells are directly involved in lung homeostasis. Still, the immune regulatory mechanisms have not been fully elucidated. Recently, studies have revealed that a variety of immune cell types, such as macrophages and T cells, are involved in the development of pulmonary fibrosis, and there is increasing evidence from idiopathic pulmonary fibrosis (IPF) and silicosis patients that T cell subsets play an essential role in the pathogenesis of pulmonary fibrosis. Patients with pulmonary fibrosis have a significantly higher proportion of T-cell subsets (1). This review summarizes the roles of CD4^+^T and CD8^+^T cells in fibrotic diseases such as IPF and silicosis, as well as related therapeutic strategies, with a view to providing new perspectives for understanding the pathogenesis of pulmonary fibrosis and developing therapeutic strategies targeting specific T cell types. T cells are commonly categorized in the following ways: by T cell receptor (TCR) type into Alpha beta (αβ) T cells and gamma delta (γδ) T cells; by co-stimulatory molecules into CD4^+^T cells and CD8^+^T cells; by function into Th cells, Regulatory T (Treg) cells, and Tc cells; and by stage of activation into initial T cells, effector T cells, and memory T cells (2).

## 2 Role and mechanisms of CD4^+^T cells in lung inflammation and fibrosis

CD4^+^T cells, immune cells that provide supportive functions for other cells of the immune system, mainly interact with antigen-presenting cells (APCs) such as macrophages, dendritic cells, and B cells and play an essential role in the activation and maturation of these cells. It has been shown that multiple CD4^+^T cells, including (T helper type 1 cell) Th1 cells, (T helper type 2 cell) Th2 cells, (T helper type 9 cell) Th9 cells, (T helper type 17 cell) Th17, Treg cells, T-follicular helper cells (Tfh) are involved in the development of lung fiogenesis (3–7).

CD4^+^T cells are a highly heterogeneous group of cells in the development of pulmonary fibrosis pathology. For example, balance between the CD4^+^T cell subsets has an essential impact on the development of lung fibrosis; Population data suggest that perivascular memory CD4^+^T cells play a pathogenic role in lethal COVID-19 pneumonia (8). On the contrary, experiments in mice and samples from humans showed that lung CCR2^+^CD4^+^T cells have an immunosuppressive effect and can reduce BLM-induced lung inflammation and lung fibrosis (9). In addition, *in vivo*, experiments in mice showed that CD103^high^ Treg cells inhibited pulmonary fibrosis induced by CD103^low^ tissue-resident pathogenic CD4^+^T cells (10). Given the different roles of different CD4^+^T cell subsets in silicotic fibrosis, regulating the immune balance between different CD4^+^T cell subsets plays an important role in alleviating lung inflammation and fibrosis.

### 2.1 Th1 cells

A growing number of studies have shown that Th1 cells exert resistance to lung fibrosis by inducing an inflammatory response in the lung through the release of pro-inflammatory cytokines IL-2, interferon-gamma (IFN-γ), tumor necrosis factor(TNF), IL-12, and IL-18 production (11–13). It has been shown that IFN-γ levels are reduced in the bronchoalveolar lavage fluid (BALF) of patients with IPF (14). Analysis of BALF from coal miners with and without silicosis exposed to coal dust showed that IFN-γ signaling was attenuated in silicosis patients (15). IL-12 induced naïve CD4^+^T cells to differentiate towards Th1 cells, producing the pro-inflammatory cytokine IFN-γ, which can inhibit fibroblast-induced collagen synthesis and attenuate fibrosis (16). Xu et al. further demonstrated that compared to BALB/c wild-type mice, T-bet gene (Th1 cell-specific transcription factor) deficient mice were unable to produce Th1 cytokines such as IFN-γ and were more susceptible to pulmonary fibrosis in bleomycin-induced pulmonary fibrosis mice (17); and further, in silicosis mice model it was found that Th1 cells could facilitate the lungs to undergo the clearance of silica particles through the secretion of IFN-γ and IL-2. This suggests that cytokines such as IFN-γ released by Th1 cells embody a defensive mechanism against external stimuli and thus play a role in resisting fibrotic pathogenesis. However, excessive inflammatory response may also lead to tissue damage. The immune-inflammatory response and tissue repair process involved in Th1 cells may continue to superimpose, leading to the occurrence and development of fibrotic pathology.

### 2.2 Th2 cells

In contrast to Th1, the current literature reveals that Th2 cells play an immunosuppressive and fibrosis-promoting role during lung inflammation and fibrosis by secreting cytokines (18, 19). Population-based data reveal that in patients with fibrotic interstitial lung disease (20), asthma patients (21) and cystic fibrosis (CF) infected by *Pseudomonas aeruginosa* (22), the proportion of Th2 cells was significantly elevated. Additionally, CCR4^+^Th2 cells in the BALF of CF patients were markedly elevated (9), and the significant rise in Th2 cell counts in the BALF of fibrotic interstitial lung disease patients was closely linked to severe declines in lung function (20). Furthermore, in mice, over-expression of the Th2 transcription factor GATA binding protein 3 was shown to promote lung fibrosis in mice (23). Th2 cells mainly produce cytokines IL-4, IL-5, and IL-13 and monocyte chemotactic protein-1 (MCP-1). Akira Saito et al. revealed that IL-4 and IL-13 promoted fibroblast proliferation and induced differentiation of fibroblasts into myofibroblasts (24). Belperio et al. found that using a neutralizing antibody to reduce IL-13 levels significantly attenuated bleomycin-induced fibrosis in the lungs of mice (25). It has been shown that in severe lung fibrosis mice, the degree of pulmonary fibrosis was more alleviated in IL-4 knockout mice compared to normal mice (26). In addition, recent studies have shown that the balance of different types of Th responses influences the pathology of pulmonary fibrosis. For example, dysregulation of the Th1/Th2 immune balance and enhancement of the Th2 immune response promote silicosis fibrosis in silicosis mice. Taken together, Th2 cells may play a negative role in the progression of pulmonary fibrosis.

### 2.3 Th9 cells

As a newly defined subpopulation of T helper cells, T helper type 9 (Th9) cells are activated in lung tissues of IPF patients and bleomycin-induced pulmonary fibrosis mice (27). Th9 cells are predominantly known for the release of the cytokine IL-9 (28), which activates dendritic cells, mast cells, CD8^+^T cells, and other target cells. Th9 cells have been involved in the pathologic processes of many diseases, such as inflammatory diseases, infectious diseases, autoimmune diseases, and cancer (28, 29). In patients with IPF, patients with CF, and SiO_2_-induced silicosis mouse models, IL-9 expression levels were significantly elevated, and over-expression of IL-9 in mice can promote collagen and fibronectin (FN) deposition in the bronchial regions of the lungs (30), whereas administration of IL-9-neutralizing antibodies to reduce IL-9 levels in mice can significantly suppress the fibroblasts activation, as well as collagen and FN deposition in the lungs (30). Fibroblast activation, as well as collagen deposition, can promote the process of lung fibrosis in mice (27, 31, 32). Further mechanistic studies showed that Th9 cells can promote pulmonary fibrosis in the following two aspects: on the one hand, Th9 cells promote fibroblast differentiation, activation, and collagen secretion by secreting IL-9; on the other hand, Th9 cells promote the differentiation of Th0 cells to Th2 cells by secreting IL-4, which accelerates the Th1/Th2 imbalance, and ultimately promotes the formation of pulmonary fibrosis (27). Therefore, Th9 cell-based immunotherapy, such as IPF, can be used as a therapeutic strategy for pulmonary fibrosis (27).

### 2.4 Th17 cells

Th17 cells are mainly characterized by the production of cytokines IL-17 and IL-22, secreted to contribute to host defense in many infectious situations but also accelerate the inflammatory process in various autoimmune diseases (e.g., rheumatoid arthritis) (33, 34). Human data suggest that IL-17A secreted by Th17 cells promotes fibroblast proliferation and myofibroblast transformation through IL-17RA-dependent signaling, thereby promoting lung fibrosis (35). Meanwhile, in silicosis mice, Th17 cells play an essential role in promoting inflammation and fibrosis in silicosis; on the one hand, Th17 cells increase the production of IL-22 and IL-1β by affecting the homeostasis of Th cell-mediated immune responses (36), and on the other hand, the production of IL-17 by Th17 cells can increase the production of IL-6, IL-8 and matrix metalloproteinases(MMPs) production, which are involved in promoting inflammatory and fibrotic processes in the lung (6, 36). A similar phenomenon can be observed in BLM-induced IPF mice, where Th17 cells and their secreted IL-17 promote BLM-induced lung inflammation and fibrosis (37); the mechanism may be the IL-17 can mediate transforming growth factor β (TGF-β) signaling to promote Extracellular matrix (ECM) deposition (38). At the same time, IL-17 can also involve in the process of epithelial mesenchymal transition (EMT) and promote fibroblast proliferation in the development of silicosis (39). IL-17A-neutralizing antibodies or IL-17A inhibitors significantly attenuated pulmonary fibrosis in silicosis mice (40) and radiation-induced lung injury and fibrosis in mice (41). Indeed, up-regulation of TRAF6 gene expression using anti-miR-125a-5p can attenuate the expression of Rorgt (Th17 cell-specific transcription factor), thereby alleviating silica-induced pulmonary fibrosis in mice (6). Taken together, Th17 cells play a key regulatory role in pulmonary inflammation and fibrosis (12, 42, 43).

### 2.5 Treg cells

The role of Treg cells in pulmonary fibrosis has been extensively studied in recent years, but the exact role is not fully understood. It has been shown that the function of Treg cells is significantly impaired in peripheral blood and BALF of patients with IPF (44). That upregulation of Treg cell expression by decreasing C-C chemokine receptor 7 (CCR7) expression can attenuate fibrosis in mice (45). These results suggest that enhancing the function of Treg cells can attenuate pulmonary fibrosis. However, some studies have also shown that the proportion and number of Treg cells are significantly increased in patients with pulmonary fibrosis (46) and that secretion of TGF-β by Treg cells induces abnormal proliferation and activation of fibroblasts, promoting the development of pulmonary fibrosis. Reduced Treg cells in mice by intraperitoneal injection of appropriate amounts of CD25 antibody can attenuate the progression of silicosis mice (47) and radiation-induced pulmonary fibrosis (48), and further mechanistic experiments showed that the CD25 antibody could reduce the accumulation of fibroblasts to attenuate pulmonary fibrosis in mice by enhancing the Th17 cell response and altering IFN-γ, IL-12/IL-4, IL-5 secretion (48). Furthermore, CD4^+^CD25^+^Treg cells can enhance the secretion of Th2 cytokines and inhibit the secretion of Th1 cytokines, which makes the Th1/Th2 immune imbalance equilibrium (49) and promote the lung fibrotic process. These results suggest that an increased proportion of Treg cells promotes the pathogenetic process of lung fibrogenesis (50, 51), and the main reason for the opposite results in the above two aspects is that (52–55): the role of Treg cells in the progression of lung fibrosis depends on the disease progression, For example, Treg cells may mediate TGF-β1 production and collagen accumulation in the early stage but may attenuate silicosis in mice in the late stage (52). Combined, Treg cells play a key role in maintaining immune tolerance and immune homeostasis, Treg cell interactions with other T-cell subsets play both antifibrotic and pro-fibrotic effects depending on the stage of pulmonary fibrosis.

### 2.6 T-follicular helper cells

T-follicular helper cells (Tfh) are characterized by the expression of a lineage-specific transcription factor (B-cell lymphoma 6, Bcl6) and the production of IL-21. It has been found that elevated levels of CXCR5^+^ICOS^+^ Programmed Death-1(PD-1)^+^ Tfh cells in the peripheral blood of IPF patients (56). In addition, CXCR5^+^ICOS^+^PD-1^+^Tfh levels were associated with systemic sclerosis (SSc). Studies have shown that administering IL-21 and inducible T-cell co-stimulators (ICOS) antibodies in mice can effectively inhibit collagen deposition and fibroblast proliferation at the skin site, thereby reducing the fibrosis process (57). As a new subpopulation of T cells, Tfh cells may bring new insights into treating fibrotic diseases.

## 3 Role and mechanisms of CD8^+^T cells in lung inflammation and fibrosis

Cytotoxic T lymphocytes (CTL cells), often called CD8^+^T cells, use CD8 glycoprotein as an identity marker and are a key component of the adaptive immune system. They play a role in the immune system’s defense against pathogens (such as viruses and bacteria) and tumors (58). CD8^+^T cells mainly secrete large amounts of IFN-γ and protease granzyme B, which work synergistically against infection or kill tumorigenic cells (59). Recent research on the single-cell analysis of relevant clinical samples shows that patients with chronic obstructive pulmonary disease (COPD) (60) and IPF (61). Patients harbored more CD8^+^T cells in the lungs which was correlated with total lung capacity and the degree of pulmonary fibrosis (62, 63). Significantly, these observations were not limited to settings of chronic viral infection but were also observed in BLM-induced fibrosis mice; the number of CD8^+^T cells was significantly increased compared with mice treated with normal saline (64). Moreover, CD8^+^T cells are sufficient to induce key hallmarks of CD8^+^T cell exhaustion; aggregated CD8^+^T cells exhibit elevated PD-1 and PD-L1 mRNA levels in IPF patients and BLM-induced fibrosis mice (65), indicating that the lung in a stage of profibrotic and proinflammatory activation (66). Population-based data suggest that CD8^+^TEMRA cells are increased in the lungs of mild-to-moderate COPD and may contribute to inflammation that precedes severe disease. Further study of these CD8^+^T cells may have therapeutic implications for preventing severe COPD (60). Meanwhile, human data suggest that smoking may damage lungs by increasing the recruitment and retention of GZMB^+^CD8^+^Trms via CXCR6 and CD103 (67). Thus, reducing CD8^+^T cells in mice by using CD8 neutralizing antibodies can reduce BLM-induced lung fibrosis in mice (68, 69). Using vitamin D3 can alleviate pulmonary fibrosis by enhancing the cytotoxic activity of CD8^+^ effector T cells in mice (70).

**Figure 1:**
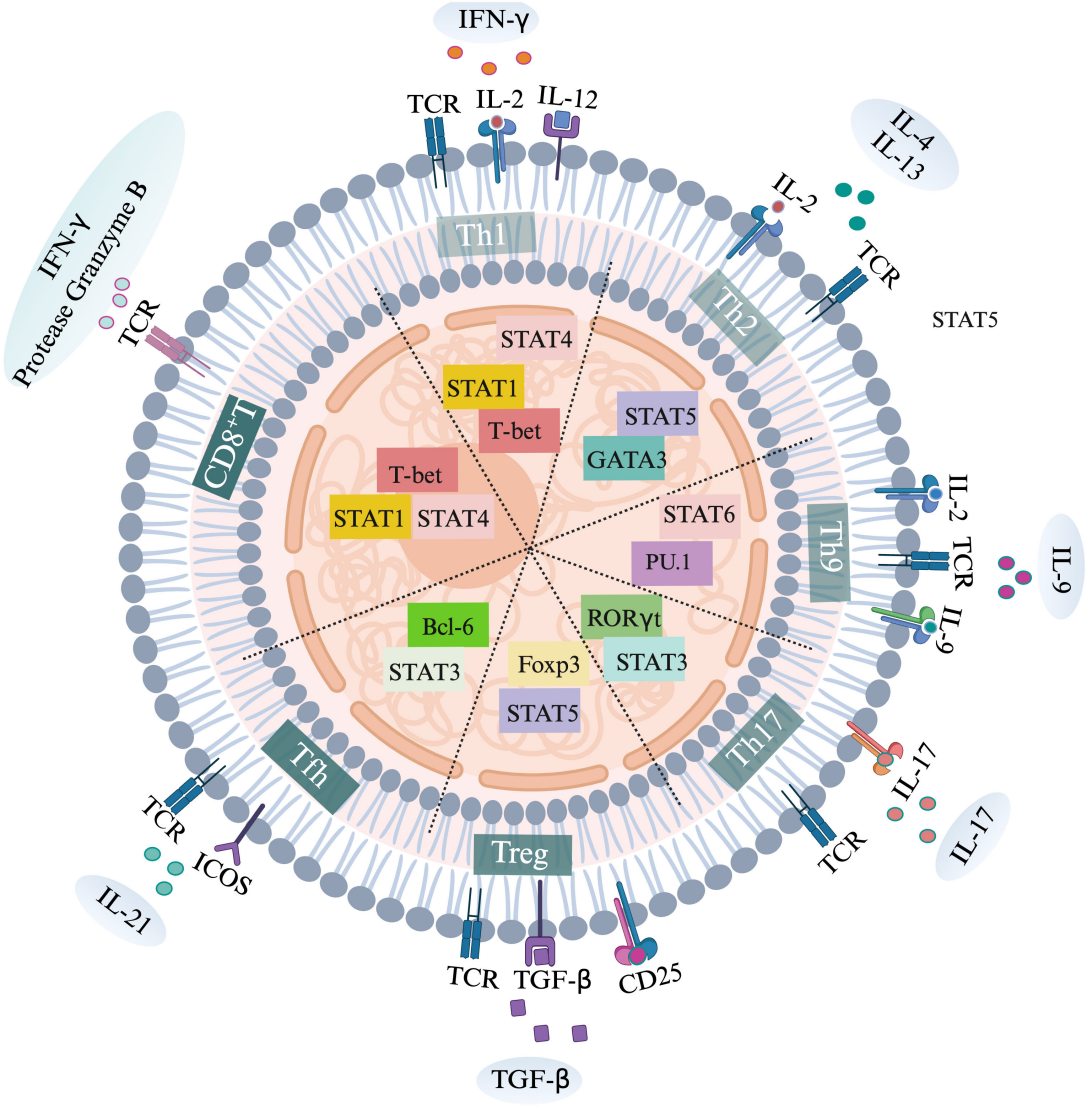
Activation of various T cell subtypes, specific transcription factors and secretion of specific cytokines.

Furthermore, some CD8^+^T cells were identified and played a vital role in the pathological process of pulmonary inflammation and fibrosis. Experiments in mice showed that maintenance of the CD4^+^tissue-resident helper T (T_RH_) cell population contributes to the differentiation of lung CD8^+^tissue-resident memory T (T_RM_) cells after influenza infection (71, 72). A new Single-cell sequencing analysis of patients with fibrotic hypersensitivity pneumonitis showed that GZMHhi CD8^+^ and GZMKhi CD8^+^T cells were significantly increased in the lung immune environment of patients with fibrotic hypersensitivity pneumonitis (73). Experiments in mice showed that blocking C-C chemokine receptor 8 (CCR8) prevented T_RM_ cell recruitment and inhibited lung fibrosis (64). Human clinical data indicate that reducing T_RM_ cells in the lungs can alleviate the progression of pulmonary fibrosis caused by acute viral infection (74). In addition, allergen-sensitized lung microenvironment interferes with the formation of antiviral resident memory CD8^+^T cells and viral clearance in the lung, thereby aggravating asthma (75). Furthermore, the reduction of CD8^+^T_RM_ cells suppressed persistent chronic pulmonary inflammation and improved tissue fibrosis in aged animals (76). Therefore, this evidence indicates that external stimuli induce the accumulation of CD8^+^T cells and some CD8^+^T cell subsets in lesions, which accelerates lung damage and leads to the formation of pulmonary fibrosis (60, 62, 77). Our study suggests disrupting the CD8^+^T cell-mediated microenvironment as a promising therapeutic strategy to enhance anti-fibrosis effects (**Figure 1**).

## 4 Role and mechanisms of other T cell subsets in lung inflammation and fibrosis

### 4.1 NKT cells

Natural killer (NK) T cells are innate cytotoxic lymphocytes that clear senescent cells. NKT cells have two distinct phenotypes: cytotoxic CD56^dim^CD16^+^ and pro-inflammatory CD56^bright^CD16^-^ (78). Peripheral NK T cells are significantly reduced in patients with idiopathic fibrosis (46). NKT cells express αβ TCR and recognize glycolipid antigens presented by the MHC I-like protein CD1δ. *In vivo* experiments in mice have shown that NKT cells attenuate bleomycin BLM-induced pulmonary fibrosis in mouse lungs by producing IFN-γ, thereby reducing collagen deposition and fibroblast activation and proliferation (79). It has been shown that the proportion and activity of NKT cells are reduced in the lungs of patients with IPF (78).

### 4.2 γδ T cells

γδ T cells are an unconventional type of T cells characterized by the fact that the TCR γ and δ chains are not restricted by the major histocompatibility complex (MHC). These cells may initiate or alleviate fibrosis in a cytokine-dependent and disease-independent manner. It has been shown that the proportion of γδ T cells is elevated in BALF from patients with IPF (80). Vγ9Vδ2, a subpopulation of human γδ T cells, induces the production of IFN-γ and shows an antifibrotic potential effect (81), meanwhile, clinical data suggest that the allogeneic Vγ9Vδ2 T can also be used to treat tuberculosis (82). Clinical data showed that γδ T cells can attenuate BLM-induced fibrosis by producing C-X-C motif chemokine ligand 10 (CXCL10) (78). Human data suggest that γδ T cells play a novel protective role in the lung during severe bacterial pneumonia by regulating excessive neutrophil-associated inflammation (83). *In vivo* experiments, IFN-γ produced by γδ T cells can attenuate BLM-induced mice of pulmonary fibrosis by indirectly inhibiting Th17 cells, thereby reducing collagen deposition as well as fibroblast proliferation (84). Modeling of wild-type mice and TCRδ-/- mice showed that lung fibrosis was more severe in BLM-induced TCRδ-/- mice, which were injected with γδT cells from WT mice, and the number of IL-17A^+^ CD4^+^T cells in the lungs of TCRδ-/- mice was lower than that of un intervened TCRδ-/- mice, suggesting that γδT cells reduced pulmonary fibrosis in mice by reducing the number of Th17 cells in the lungs reduced the process of BLM-induced pulmonary fibrosis (84) (**Table 1**).

**Table 1:** Classification of T cell subtypes and their role in lung inflammation and fibrosis.

T cell subtypes	Annotation	Species	Disease	Effect in fibrosis	Mechanism	References
CD4^+^T	Th1	Human	Silicosis	Anti-fibrosis	inducing the production of IFN-γ	(15)
	Th1	Mouse	Silicosis	Anti-fibrosis	IFN-γ released by Th1 cells embody a defensive mechanism against external stimuli and thus play a role in resisting fibrotic pathogenesis	(17)
	Th2	Mouse	Pulmonary fibrosis	Pro-fibrotic	IL-4 and IL-13 produced by Th2 cells can promote fibroblast proliferation and induced differentiation of fibroblasts into myofibroblasts	(24, 25)
	Th9	Mouse	Silicosis	Pro-fibrotic	promoting fibroblast differentiation, activation, and collagen secretion by secreting IL-9; promoting the differentiation of Th0 cells to Th2 cells by secreting IL-4, which accelerates the Th1/Th2 imbalance, and ultimately promotes the formation of pulmonary fibrosis	(27, 30)
	Th17	Mouse	Silicosis	Pro-fibrotic	IL-17 can mediate transforming growth factor β (TGF-β) signaling to promote Extracellular matrix (ECM) deposition	(37, 40)
	Treg	Mouse	Silicosis	Pro-fibrotic(early) Anti-fibrotic(late)	mediating TGF-β1 production and collagen accumulation(early) inducing the activation and proliferation of fibroblasts and deposition of collagen (late)	(52)
	CD103^high^ Treg	Mouse	Aspergillus antigen induced fibrosis	Anti-fibrotic	constraining the ability of pathogenic CD103lo T_RM_ cells to cause fibrosis	(10)
	Tfh-	Mouse	IPF	Pro-fibrotic	inducing the deposition of collagen and proliferation of fibroblast	(56)
	CCR2^+^CD4^+^T	Human	BLM induced fibrosis	Anti-fibrotic	inducing the deposition of collagen and proliferation of fibroblast	(9)
	LAG3^+^CD4^+^T	Human	Systemic sclerosis-associated interstitial lung disease	Anti-fibrotic	inducing the production of TGF-β1	(85)
CD8^+^T	cytotoxic CD8^+^effector T	Human	Pulmonary fibrosis	Anti-fibrotic	inducing the production of IFN-γ and protease granzyme B	(59)
NK T	cytotoxic CD56^dim^CD16^+^ NK T cells	Mouse	BLM-induced fibrosis	Anti-fibrotic	inducing the production of IFN-γ	(78)
γδ T	γδ T- Vγ9Vδ2	Human	Systemic sclerosis	Anti-fibrotic	inducing the production of IFN-γ	(81)
	γδ T	Human	BLM-induced fibrosis	Anti-fibrotic	inducing the production of IFN-γ inhibiting the function of Th17	(78)

## 5 Current strategies for treating pulmonary fibrosis by targeting and regulating CD4^+^T cells and CD8^+^T cells

### 5.1 Gene therapy that regulate CD4^+^T cells or CD8^+^T cells in lung fibrosis

TCR: The primary role of the TCR is the first signal of T cell activation transduction; TCR can be generated by binding to the MHC of APCs, thereby generating the first signal of T cell activation. TCR signaling synergistically interacts with cytokines, co-stimulatory molecules, chemokines, integrins, and metabolite-induced signaling pathways to drive the activated T cells to differentiate into specific T cell subtypes (86). It has been shown that lung fibrosis modeling in mice with BLM showed a significant increase in interstitial inflammation and collagen deposition as well as a significant delay in epithelial cell regeneration in γδ T TCR knockout mice compared to wild-type mice (87), suggesting that the γδ T TCR may be capable of reducing lung fibrosis in mice. The bleomycin mouse model showed that TCRδ–/– deficient mice developed more severe pulmonary fibrosis than wild-type mice (84).

CD28: CD28 acts as a second signal of T-cell activation, which amplifies the first signal of TCR transmission. It has been shown that CD8^+^ CD28^-^T cells are significantly increased in IPF patients compared to normal lung explants. CD28^-^T cells in IPF patients may promote lung fibrosis, but the immune checkpoint proteins Cytotoxic T-Lymphocyte-Associated Antigen-4 (CTLA-4) and PD-1 limit this effect (66), and it has also been shown that the poor prognosis of IPF patients is associated with downregulation of CD28 on CD4^+^ T cells in the periphery system (88, 89).

OX40: OX40 (also known as CD134, Tumor necrosis factor receptor superfamily, member 4) and its binding partner OX40 ligand (OX40L; also known as CD252) are members of the TNF. TNF receptor superfamily is expressed on activated CD4^+^T and CD8^+^T cells and many lymphocytes and non-lymphocytes (90). OX40L is primarily expressed by antigen presenting cells (APCs) such as dendritic cells, but B cells, macrophages, and Langerhans cells also express OX40L (91). Epidemiological data suggest that dendritic cells expressed OX40L in the respiratory airways in response to epithelial cell-derived thymic stromal lymphopoietin (TSLP) (92). *In vivo* experiments in a mice asthma model showed that OX40L inhibitors suppressed TSLP-mediated Th2 inflammation and reduced the number of OX40L^+^dendritic cells in the lungs (93). Indeed, the use of OX40L antibodies can effectively inhibit the proliferation of CD4^+^OX40^+^T cells in the ovalbumin-induced asthma mouse model and may further reduce the inflammatory response by altering the secretion of IL-4, IL-6, IL-13, IL-17, and TNF-α, and IFN-γ, and thus alleviate the progression of asthma (94). OX40L is encoded by the TNFRSF4 and TNFSF4 genes. Studies have shown that establishing a skin fibrosis model in mice using BLM showed that compared with wild-type mice, Tnfsf4-/- mice had lower inflammation, less inflammatory cell infiltration, and less fibrosis in the lesion site (95). These studies suggest blocking OX40/OX40L can reduce lung inflammation and fibrosis. The OX40/OX40L interaction has been shown to play a central role in developing many inflammatory and autoimmune diseases, suggesting that they may be suitable candidates for clinical intervention (96).

PD-1: PD-1 (encoded by Pdcd1) is a co-suppressor receptor on T cells that signals PD- L1 and PD- L2 through its ligands. It has been shown that PD-1 and PD-L1 are over-expressed by CD4^+^T cells in the peripheral blood of patients with IPF relative to normal subjects (97). It has been demonstrated that upregulating PD-1 gene expression in CD4^+^T cells from IPF patients can promote the process of lung fibrosis (98), and experiments carried out in mice have shown that up-regulation of the PD-1 gene promotes the process of silicosis fibrosis (99). In a mouse model of BLM chemical injury, PD-1 was upregulated on CD8^+^T cells in the lungs, suggesting in pulmonary fibrosis, an immunosuppressive environment that blockades of IL-6, CD47, and PD-L1 leading the process of lung fibrosis reversed (100), A study of normal individuals and individuals with tuberculosis showed a significant increase in PD-1^+^CD4^+^T cells in individuals with tuberculosis relative to normal individuals (101), Gancao Ganjiang decoction (GGD) (A traditional Chinese medicine prescription consisting of liquorice and dried ginger) was found to increase body weight and decrease lung index in mice with pulmonary fibrosis, GGD significantly ameliorated inflammation and attenuated IPF in lung tissue of mice. GGD treatment reduced the levels of PD-1 in lung tissues and the expression of PD-1 in peripheral blood CD4^+^T cells of mice with IPF (102). Intraperitoneal administration of PD-1/PD-L1 inhibitors to mice inhibits fibroblast activation and collagen secretion by fibroblasts, thereby slowing down the progression of lung fibrosis in mice (103).

CD27: CD27 is a TNF receptor-associated factor (TRAF)-linked receptor expressed only on lymphoid cells, including T cells, B cells, and NK cells. Expression of CD27 is decreased in peripheral blood CD4^+^T cells in IPF patients, and downregulation of CD27 is associated with lung function dysfunction. Notably, CD27 can suppress fibroblast transformation to myofibroblast and ECM production. T-cell-derived soluble CD27 significantly reduces fibroblastic collagen and fibronectin synthesis (104). Furthermore, studies using blood and BAL cells from patients with bleomycin-induced disease have shown that loss of CD27 on lung T cells correlates with the severity of lung inflammation (105). In addition, CD70 is also expressed on the surface of fibroblasts and is regulated by cell density and TGF-β1. Moreover, It has been shown that up-regulation of CD27 activity on human T cells precisely activate CD70 activity on fibroblasts to reduce collagen deposition and inhibit pulmonary fibrosis by reducing fibroblast proliferation (104).

ICOS: Inducible T-cell co-stimulators (ICOS) is a new member of the immunoglobulin (Ig) co-receptor molecule family. Recently, studies have focused on the role of ICOS on adaptive immune responses in lung fibrosis (106). High expression of ICOS on the surface of CD4^+^T cells in IPF patients is associated with improved survival (89).

CD137 (also called 4-1BB, TNF receptor superfamily member 9): (TNF receptor superfamily 9) is an inducible co-stimulatory receptor expressed on activated T cells and NK cells (107). Blocking the 4-1BB pathway by intraperitoneal injection of 4-1BB antibody in mice with silicosis reduces the Th1, Th17 ratio in the lung tissues of mice with silicosis and thus reduces fibroblast aggregation and collagen deposition in the immune microenvironment of the lungs, thereby alleviated the progression of silica-induced pulmonary fibrosis in the lungs of mice (108) (**Table 2**).

**Table 2:** Current strategies for treating pulmonary fibrosis by targeting and regulating CD4^+^T cells and CD8^+^T cells.

Category	Molecule/Receptor	Target	Manifestations in pulmonary fibrosis	Preclinical/Already clinically tested
**T cell activation**	TCR (First signal)	MHC(APCs)	The silicosis mouse model established with bleomycin showed that γδ T TCR knockout mice exhibited more severe pulmonary fibrosis compared with normal mice.	Preclinical
	CD28 (Second signal)	CD80(APCs)	IPF patients have increased CD8^+^CD28^-^T cells in the lung immune microenvironment compared with normal people, and downregulation of CD28 in CD4^+^T cells is associated with poor prognosis.	Preclinical
**Inflammatory regulation**	OX40	OX40L(APCs)	*In vivo* experiments in mice showed that blocking OX40/OX40L binding could reduce lung inflammation and fibrosis.	Preclinical
	PD-1	PD-L1 PD-L2(APCs)	Overexpression of PD-1/PD-L1 promotes fibrosis in IPF patients.	Preclinical
**Fibroblast regulation**	CD27	CD70(fibroblasts)	Downregulation of CD27 in IPF patients is associated with lung dysfunction. Upregulating CD27 activity on human T cells and thereby enhancing CD70 activity on fibroblasts can promote fibroblast apoptosis, thereby reducing collagen deposition and inhibiting pulmonary fibrosis.	Preclinical
	4-1BB	4-1BBL(APCs)	Blocking the 4-1BB pathway in mice can alleviate the progression of silicosis fibrosis by reducing the ratio of Th1/Th17 cells.	Preclinical
**Survival and immune response**	ICOS	ICOSL(APCs)	High expression of ICOS on the surface of CD4^+^T cells in IPF patients is associated with improved survival.	Preclinical

## 6 Related drug research by targeting CD4^+^T cells or CD8^+^T cells in lung fibrosis

Neutralizing Antibodies: Knockdown of miR-125a-5p gene in mice with silicosis by administration of miR-125a-5p antibody slows down silica-induced pulmonary fibrosis in mice by regulating T-cell subset occupancy (6); a model of dermatofibrosis in mice with hypochlorite showed that acazicolcept reduced inflammatory cells infiltration in the skin lesions and decreased fibrosis levels in the spleen and lung by decreasing CD69 and PD-1 expression of CD4^+^T cells, as well as reducing fibrosis in the spleen and lung, as compared with Fc-control mice. Acazicolcept can significantly reduce inflammatory cell infiltration and fibrosis in mouse skin lesions by decreasing CD69 and PD-1 expression of CD4^+^T cells in the spleen and lungs of mice (109).

Traditional Chinese Medicine (The collection, processing and preparation of drugs are guided by traditional Chinese medical theories): Experiments in mice showed that dihydrotanshinone I can alleviate crystalline silica-induced lung inflammation by weakening Th1, Th2, Th17, and Treg immune responses in the mouse lungs and inhibiting fibroblast activation and proliferation as well as collagen deposition (110), N-acetylcysteine prevents pulmonary fibrosis in a mouse silicosis model by modulating CD4^+^T cells immune responses and thereby reducing collagen deposition (111), Dehydrozingerone (DHZ) is an analog of curcumin. The study results showed that DHZ has an anti-fibrotic effect on pulmonary fibrosis by regulating Wnt/β-catenin signaling. DHZ administration attenuated BLM-induced increase in lung index, inflammatory cell infiltration, and hydroxyproline(HYP) levels in lung tissues (112). Experiments conducted in Rats showed that Yang Fei Huoxue Decoction (YHD) reduced the expression of CD28, CD80, CD86, Delta-like1, Notch2 and Notch3 while upregulating the ratios of Th1/Th2 and Tc1/Tc2, thereby reducing the degree of alveolar inflammation and fibrosis in mice (113). Data from the human population indicate that oral administration of bacterial lysate OM-85(Bacterial Lysates )can slow down pulmonary fibrosis by restoring the Th1/Th2 immune balance (114). 

**Figure 2:**
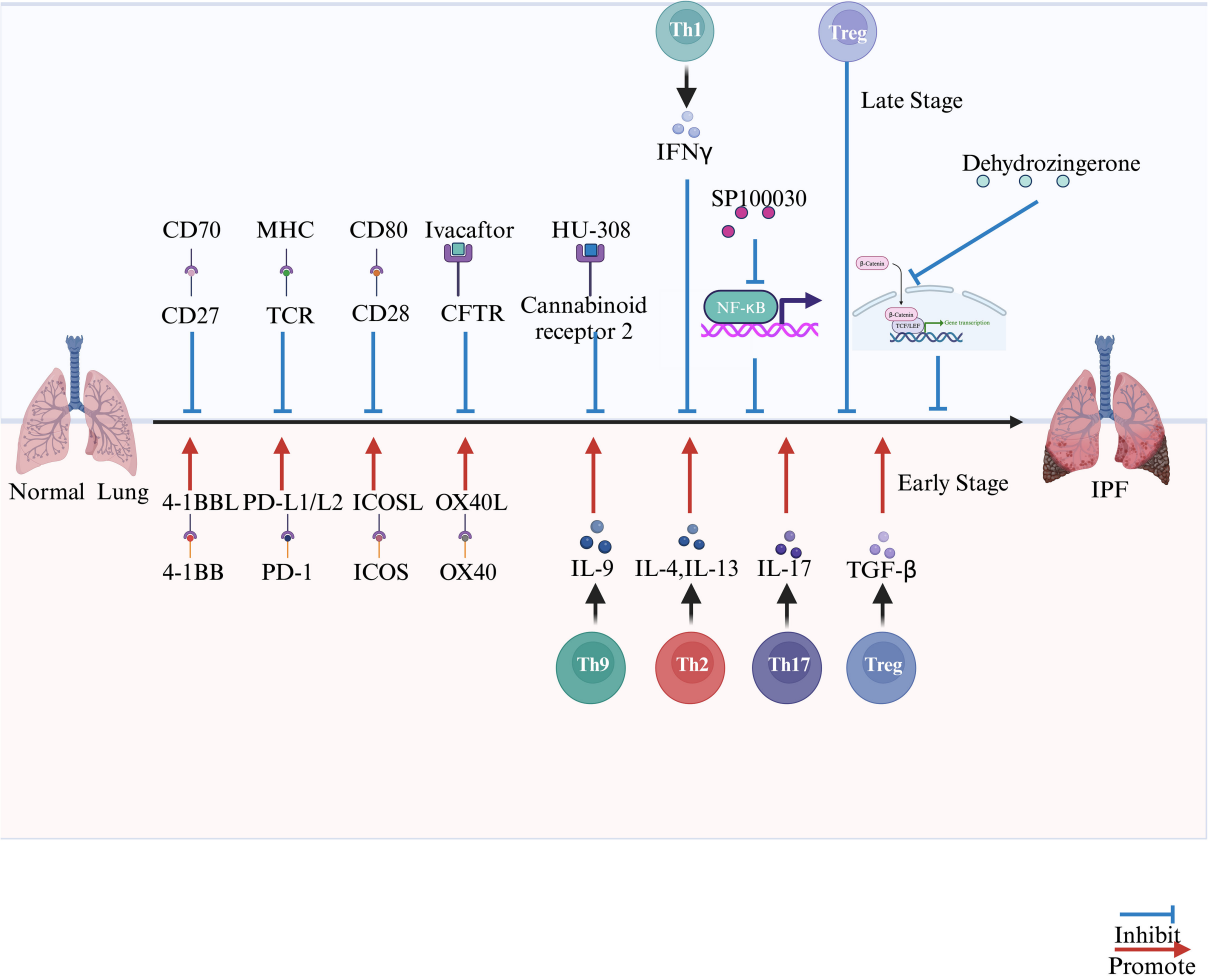
The role of T cell and T cell receptor in the progression of pulmonary fibrosis.

Western medicine (Drugs made by chemical synthesis or extracted from natural products): Patients with CF have significantly reduced populations of circulating CD4^+^T cells and CD8^+^T cells, CD19^+^B cells, and CD16^+^CD56^+^NKT cells in their bloodstreams compared to healthy controls and elexacaftor-tezacaftor -ivacaftor triple therapy moderately increased their levels (115), pirfenidone (PFD) attenuated silica-induced inflammation and fibrosis in mouse lungs by inhibiting IL-17A secretion (116), and bosutinib therapy reduced CD4^+^T cells, as well as CD8^+^T cells, by decreasing CD4^+^T cells, CD19^+^B cells, and CD16^+^CD56^+^natural killer cell populations in mouse lung tissues (117). HU-308 (specifically binds to Cannabinoid receptor 2) attenuates lung fibrosis by inhibiting BLM-induced polarization of mouse Th2 cells through the specific activation of Cannabinoid Receptor 2 (CB2) on CD4^+^T cells (118). The cholinesterase inhibitor donepezil attenuates lung fibrosis in mice by inhibiting the activation of Th17 cells and inhibiting fibroblast activation and collagen deposition (119). Li et al. conducted animal experiments to illustrate that silica stimulates the body to produce cytokines that promote the proliferation of Effective Memory T cells (T_EM_), while tauroursodeoxycholic acid reduces the increase of pathogenic T_EM_ cells in the lungs of mice, thereby inhibiting the activation and proliferation of fibroblasts and collagen deposition, which can slow down pulmonary fibrosis (120) (**Figure 2**, **Table 3**).

**Table 3:** Current drugs targeting T cell markers and signaling pathways to slow lung fibrosis and inflammation.

Drug	Target	Mechanism	Disease	References	Targeting T cells	Species
Medicine						
Ivacaftor	CFTR	CFTR agonist	Cystic fibrosis	(115)	CD4^+^T cells CD8^+^T cells	Human
Donepezil	Th17 cells	α7nAchR-JAK2-STAT3 pathway inhibitor	Pulmonary fibrosis	(119)	Th17 cells	Mouse
AK-7	SIRT-2	SIRT-2 inhibitor	Pulmonary Inflammation	(121)	CD4^+^T cells CD8^+^T cells	Mouse
Dihydrotanshinone I	STAT1 STAT3	STAT1 STAT3 inhibitor	Pulmonary Inflammation	(110)	Th cells	Mouse
Tauroursodeoxycholic acid	Tem cells	Tem cells inhibitor	silicosis	(120)	T effector memory cells	Mouse
Pathway Inhibitors						
SP100030	Nuclear Factor-kB	Nuclear Factor-κB inhibitor	Pulmonary fibrosis	(128)	T cells	Mouse
Pirfenidone	JAK-STAT	JAK inhibitor	Pulmonary fibrosis	(116)	Th17 cells	Mouse
Dehydrozingerone	Wnt/β-catenin	Wnt/β-catenin inhibitor	pulmonary fibrosis	(112)	T cells	Mouse
Gancao Ganjiang decoction	PD-1	PD-1 inhibitor	Idiopathic pulmonary fibrosis	(102)	Th17 cells	Mouse
HU-308	Cannabinoid receptor 2	JAK/SOCS3 signaling	Systemic sclerosis	(118)	Th2 cells	Mouse

## 7 Potential signaling pathways by targeting CD4^+^T cells or CD8^+^T cells in lung fibrosis

TGF-β Signaling Pathway: TGF-β is a member of a large family of polypeptides involved in the regulation of a variety of biological processes, including proliferation, differentiation, and apoptosis. The development of lung fibrosis is inextricably linked to the TGF-β signaling pathway, which is activated during the development of lung fibrosis (122), and its activation results in the activation of a variety of cell types such as macrophages (123), platelets (124) and T cells, among others, to synthesize increased TGF-β, resulting in a positive cascade reaction that increases ECM production. Evidence from *in vivo* experiments in mice suggests that TGF-β significantly affects T lymphocytes’ proliferation, activation, and function and that the pathological progression of pulmonary fibrosis in mice can be modulated by regulating the TGF-β signaling pathway on T lymphocytes (125). Studies in mice have found that TGF-β3 secreted by CD4^+^LAG3^+^T cells inhibits TGF-β1-induced mesenchymal transformation, regulates cell function, and reduces collagen release (85). The lymphocyte-specific protein tyrosine kinase-specific inhibitor A-770041 attenuates lung fibrosis by inhibiting TGF-β production in regulatory T cells (126). Studies from clinical samples have shown that upregulation of PD-1 signaling on CD4^+^T cells promotes the process of lung fibrosis by facilitating signal transducer and activator of transcription 3 (STAT3)-mediated TGF-β1 production (98), and these results suggest that TGF-β can influence the development of lung fibrogenesis by modulating the alteration of T cells from a latent to an activated state.

Nuclear Factor-κB Signaling Pathway: The nuclear factor κB (NF-κB) family of transcription factors has been implicated in various inflammatory diseases (127). *In vivo* experiments in mice have shown that AK-7 targets SIRT-2 by inhibiting the NF-κB signaling pathway on T cells, thereby inhibiting airway inflammation and oxidative damage, slowing down collagen deposition and pulmonary fibrosis in COPD mice (121), and NF-κB inhibitor SP100030 inhibited the activity of T cells could reduce the immune microenvironment inflammation and also could upregulate fibroblast activation, proliferation, and collagen deposition in lungs of mice (128). In conclusion, inhibition of the NF-κB signaling pathway on T cells can reduce inflammation and fibrosis in the lung.

Notch Signaling Pathway: The Notch signaling pathway is closely related to T cell immune function and is involved in the development, differentiation, and activation of a variety of T cells. Notch is involved in lung fibrosis by regulating the homeostatic state of T cells and cytokine expression. Experiments carried out in mice showed that when the Notch signaling pathway was inhibited using the Notch inhibitor Dual-anti platelet-therapy (DAPT), it reversed the Th1/Th2 ratio imbalance, increasing the Th1 ratio (129), and the activation of various receptors and ligands in the Notch signaling pathway also had different effects on CD4^+^T cell differentiation, wherein, Dll1 induced Th1 cells differentiation and Jagged induces Th2 cells differentiation (130). Notch signaling pathway proteins Notch1, Notch2, Jag1, Jag2, DLL1, and DLL4 were significantly upregulated in lung tissues of the rat pneumonia animal model compared to control rats. Meanwhile, the Th1/Th2 ratio in BALF was reduced in the model rats, and inhibition of the Notch signaling pathway by DAPT reversed the imbalance of the Th1/Th2 ratio. Therefore, blockade of the Notch pathway can reverse the Th1/Th2 imbalance in the inflammatory response of the lungs and inhibit pulmonary fibrosis (109). Therapeutic agents targeting the Notch pathway, including γ-secretase inhibitors and dendritic cell immunotherapy, have been shown to be effective in different models of airway inflammatory disease.

Wnt Signaling Pathway: Wnt signaling plays a key role in tissue homeostasis and cell fate decisions in animal embryos and post-embryonic development (131, 132). Some Wnt signaling pathway key components are essential in developing normal T cells. Epidemiological data have shown that secreted frizzled-related protein 2 (SFRP2, can bind to FZD receptor to regulate Wnt signaling) can promote COPD airway inflammation and Th17/Treg imbalance through the Wnt/β-catenin pathway (133). Indeed, T cell factor (TCF) 1(downstream transcription factors of Wnt/β-catenin signaling) was required for normal thymic T cell development (134), plays a critical role in the generation and persistence of functional memory CD8^+^T cells, as well as in the activation of CD4^+^T cells to promote Th2 differentiation and inhibit Th17 differentiation. Less IFN-γ production from CD4^+^T cells from TCF-1-deficient mice compared with CD4^+^T cells from wild-type mice (135). Moreover, activated β*-*catenin (transcription factors of Wnt/β-catenin signaling) promoted the formation of CD8^+^ memory T cells and enhanced the protective capacity of CD4^+^CD25^+^ regulatory T cells. And blocking the Wnt/β-catenin signaling pathway by direct oral-bronchial injection of β-catenin shRNA in silicosis mice could alter the CD4^+^T subpopulation and could alleviate the process of silica-induced lung fibrosis (3). In addition, several Wnt ligands have been demonstrated to be essential for T cell development. Wnt4 and Wnt5a are not dependent on β-catenin, and their effects on T cell development appear opposite, with Wnt4 enhancing thymopoiesis and Wnt5a promoting apoptosis in thymocytes. Wnt10b deficiency mice of allergic asthma results in enhanced memory T cell activation, increased Th2 polarization as evidenced by elevated IL-4 and IL-13 concentrations, and recombinant Wnt10b increases the percentage of naïve CD4^+^T and CD8^+^T cells *in vitro*, thereby mitigating allergic asthma (136). These new findings suggest that modulating Wnt pathway activity may effectively enhance protective immunity and treat autoimmune diseases (137).

PI3K/AKT Signaling Pathway: The phosphatidylinositol 3-kinase (PI3K)/protein kinase B (PKB/AKT) signaling pathway is one of the core signaling pathways in cells that regulates cell growth, proliferation, movement, metabolism, and survival (138). There were no significant differences in the expression of PI3K p110 α, β, and δ isoforms in normal and IPF tissue/tissue-derived fibroblasts, whereas p110γ was more expressed in IPF lung homogenates and ex vivo fibroblast cell lines. Significantly, both pharmacological inhibition and gene silencing of p110γ can dramatically inhibit the proliferation rate and α-smooth muscle actin (α-sma) expression of IPF fibroblasts (139). Studies have shown that *in vivo* experiments in mice inhibit the PI3K/AKT signaling pathway and alleviate pulmonary fibrosis by reducing the activation and proliferation of fibroblasts (138). Some evidence revealed that inhibition of p110δ restored inflammatory responses against a variety of fibrosis. For example, Leniolisib (PI3Kδ inhibitors) may affect T cell redistribution and function (140). PI3Kδ knockout mice have less production of IFN-γ, granzyme B, and perforin derived by CD8^+^T cells (141–143) compared with wild-type mice. Experiments in mice showed that mice with inactivated p110δ were able to reduce the hyperrespiratory response induced by inhaled methacholine (144).

## 8 Between CD4^+^T and CD8^+^T and the pulmonary microenvironment in lung inflammation and fibrosis

### 8.1 CD4^+^T cells alleviate pulmonary fibrosis by regulating apoptosis of epithelial cells and fibroblasts

#### 8.1.1 CD4^+^T cells and epithelial cells

In chronic obstructive pulmonary disease (COPD), asthma, and infectious lung diseases, the interaction between CD4^+^T cells and epithelial cells play a critical role, with distinct mechanisms and manifestations observed across these conditions. Research has indicated significant infiltration of CD4^+^T cells in bronchial biopsies and lung tissue samples from patients with COPD and asthma (145). In COPD, the chemokines CXCL10 and CXCL12, secreted by epithelial cells and macrophages, activate CXCR3 on the surface of Th1 cells. This activation prompts the release of interferon-gamma (IFN-γ). While IFN-γ exhibits anti-inflammatory properties, it can simultaneously inflict inflammatory damage on lung epithelial cells. In asthma, damaged epithelial cells release IL-33, which stimulates Th2 cells by way of Th2 inflammatory cytokines from CD4^+^T cells (146). Activated Th2 cells secrete IL-5 and IL-13, which drive eosinophilic inflammation. IL-5, in particular, triggers the release of acidic proteases that impair mucus secretion and harm epithelial cells (147, 148). Moreover, Cytokines released from Th2 T cells are crucial in infectious lung diseases including bacterial pneumonia, viral pneumonia, and infectious respiratory distress syndrome. IL-6, secreted by Th2 cells enhances Notch4 expression in Treg cells, suppressing the production of regulatory proteins and intensifying pulmonary inflammation (149). COPD is also characterized by a marked increase in Th17 cells within lung tissues, resulting in the secretion of IL-17A and IL-22.These cytokines activate cascades of proteases and lysosomal enzymes, compromising the epithelial barrier and causing epithelial cell damage. Through these mechanisms, CD4+ T cells release inflammatory mediators that overstimulate mucus production, weaken the epithelial barrier, and drive inflammation and fibrotic processes in the lungs.

#### 8.1.2 CD4^+^T cells and fibroblasts

Studies on population data have revealed that cytotoxic CD4^+^T lymphocytes may exacerbate tissue repair processes by inducing apoptosis in cells such as fibroblasts and endothelial cells, ultimately leading to fibrosis and organ dysfunction (150). Furthermore, *in vivo* mouse studies demonstrated that PD-1^+^Th17 cells activate and proliferate lung fibroblasts, driving collagen deposition through the secretion of STAT3-dependent IL-17A and TGF-β1. Notably, inhibiting PD-1 using antibodies or chemically targeting STAT3 has been shown to alleviate fibrosis (98). Furthermore, animal studies indicate that lung antigen-presenting cancer-associated fibroblasts (apCAFs) can directly activate CD4^+^T cells via MHC II presentation and secrete C1q, This secretion inhibits CD4^+^T cell apoptosis and bolsters anti-tumor immune responses (151).

### 8.2 CD8^+^T cells alleviate pulmonary fibrosis by regulating apoptosis of epithelial cells and fibroblasts

#### 8.2.1 CD8^+^T cells and epithelial cells

CD8^+^T cells induce apoptosis of lung epithelial cells primarily through three key mechanisms: perforin/granzyme B, Fas-FasL, and TNF-α. In COPD, cytotoxic CD8^+^T cells intensify epithelial damage by releasing cytotoxic substances, such as perforin and granzyme, which ultimately cause cell death (152). Interestingly, research has demonstrated that CD103^+^CD8^+^T cells, generated within the epithelial environment, can mitigate epithelial barrier damage caused by excessive cytotoxicity (152). Mouse experiments further revealed that apoptosis of epithelial cells in the lungs is associated with the upregulation of Fas and FasL expression in CD8^+^T cells (151). Additionally, *in vitro* studies indicate that human CD8^+^T cells can promote epithelial cell damage and apoptosis by secreting TNF-α (153). The role of CD8^+^T cells varies across different inflammatory responses and fibrotic disease processes. During bacterial pneumonia, CD8^+^T cells release IFN-γ following acute lung injury, which aids alveolar repair and regeneration by enhancing the activity of type II alveolar epithelial cells (154). However, in severe influenza A virus (IAV) infections, CD8^+^T cells produce both TNF-α and IFN-γ, disrupting the epithelial barrier and exacerbating IAV symptoms (153).

Under normal physiological conditions, lung epithelial cells maintain microenvironmental stability and collaborate with T cells to ensure effective immune defense. In idiopathic pulmonary fibrosis, abnormal epithelial cells can chronically activate the adaptive immune response via the internal cGAS-STING pathway. This persistent activation state enhances CD8^+^T cell activity, disrupting their normal physiological function and potentially aggravating tissue damage and fibrosis (155).

#### 8.2.2 CD8^+^T cells and fibroblasts

Cytotoxic CD8^+^T cells, recognized for their ability to eliminate virus-infected and tumor cells through both membrane-bound and soluble cytotoxic mechanisms, may also influence fibroblast activity (51). For instance, experiments in mice showed that CD8^+^T can promote apoptosis of fibroblasts (156). However, evidence from human studies (157) and *in vivo* experiments in mice (158) revealed that CD8^+^T cells can produce CCL3 (MIP-1), a chemokine associated with fibroblast recruitment to the lungs, and correlate with the development of lung fibroblasts in animal models.

Furthermore, the reciprocal effects between CD8^+^T cells and fibroblasts are multifaceted, Population studies have shown cancer-associated fibroblasts facilitate the progression of lung adenocarcinoma by suppressing the recruitment, infiltration, and cytotoxic activity of CD8^+^T cells within the lung immune microenvironment (159). Furthermore, mouse experiments have shown that fibroblasts may stimulate Th17 differentiation, induce CD8^+^T cell apoptosis, and recruit T regulatory (Treg) cells by forming an autocrine loop involving IL-6 and TGF-β, thus exacerbating lesions like vocal cord leukoma (160). Specifically, Notably, *in vitro* experiments further reveal that cancer-associated fibroblasts trigger CD8^+^T cell apoptosis through the Fas (expressed on CD8^+^T cells) and FasL (expressed on cancer-associated fibroblasts) pathways and inactivate CD8^+^T cells through the PD-1 (expressed on CD8^+^T cells) and PD-L1 (expressed on cancer-associated fibroblasts) pathways (161). In summary, the interactions between CD8^+^T cells and fibroblasts in inducing apoptosis are intricate and highly dynamic.

### 8.3 Between pulmonary microenvironment and T cell in lung fibrosis

Changes in the local immune microenvironment play a pivotal role in the progression of pulmonary fibrosis. Research reveals that CD4^+^T cells are essential for the expansion of other antigen-specific immune cells. A preliminary study on lung cancer patients (162) and individuals with HIV-related COPD (163) observed a marked depletion and dysfunction of CD4^+^T cells in the lung mucosa, accompanied by a prominent CD8^+^T cell alveolitis immune phenotype. Following retroviral therapy, HIV-infected patients exhibited a significant increase in CD4^+^T cells in both the lungs and peripheral tissues, while CD8^+^T cell numbers declined, leading to an elevated CD4/CD8^+^T cell ratio. This suggests that treatment can restore the viral immune response of CD4^+^T cells within the lung mucosa and mitigate CD8^+^T cell-driven pulmonary inflammation in patients with HIV-related COPD.

In comparison to peripheral and lymphatic tissues, the lungs are among the few anatomical sites where CD8 lineage lung-resident memory T cells (T_RM_) are relatively short-lived and do not permanently reside in the tissue. After respiratory viral infection or local immunization, CD8^+^T_RM_ cells exhibit a protective effect against lung injury caused by the same pathogen. However, following viral pneumonia, they lose homeostasis, migrating to peripheral tissues and even lymph nodes. This migration results in the depletion of CD8^+^T_RM_ cells in lung tissue and contributes to the pathogenesis of lung diseases (71). Consequently, regulating the differentiation of CD4^+^T and CD8^+^T cells is critical for maintaining the lung microenvironment and immune balance. Beyond their roles in immune homeostasis, the interplay between CD4^+^T cells, CD8^+^T cells, and macrophages in the lungs is central to alveolar regeneration and the development of pulmonary fibrosis. An increase in CD4^+^T cells in the peripheral blood of patients with idiopathic pulmonary fibrosis (IPF) may promote macrophage infiltration in the pulmonary bronchi and their subsequent polarization into the M1 phenotype. Notably, the proportion of activated CD4^+^ memory T cells and M1-like macrophage infiltration correlates positively with the alveolar-arterial oxygen pressure difference (A-aDO2) in such patients (164).

Moreover, studies indicate that during the onset and progression of acute viral pneumonia-induced fibrosis, IFN-γ and TNF derived from CD8^+^T cells stimulate macrophages to release IL-1β gradually. This leads to impaired differentiation of epithelial progenitor cells, subsequently inducing pulmonary fibrosis (165, 166). These findings underscore the importance of regulating the differentiation and interaction of CD4^+^T cells, CD8^+^T cells, and macrophages in preserving lung immune homeostasis and preventing fibrosis.

## 9 Summary

Although the pathogenesis mechanism, prevention and treatment of pulmonary fibrosis have already been partially elucidated, recently still no effective treatment of pulmonary fibrosis (167). Lung transplantation is an effective method for treating pulmonary fibrosis but has its limitations (168, 169). Many literature studies have shown that targeting CD4^+^T cells 4 (170) and CD8^+^ T cells (171) has great potential for treating pulmonary fibrosis. By producing specific CD4^+^T and CD8^+^T cell subtypes, the immune system can fine-tune itself to prevent inappropriate activation, but the role of various CD4^+^T and CD8^+^T lymphocyte subsets in the progression of pulmonary fibrosis was controversial (54). CD4^+^T cells including helper T cells 1 (Th1), Th2, Th17, follicular helper T cells, and regulatory T cells (Treg). CD4^+^T cell subtype differentiation must achieve a delicate balance to effectively respond to the invasion of infectious agents while preventing autoimmune reactions caused by excessive activation of the immune response (172). An unbalanced Th1/Th2 immune response has long been considered at the core of the pathogenesis of pulmonary fibrosis (16). Experiments in animals have shown that reducing the number of CD8^+^T cells can reduce lung inflammation and fibrosis levels. Importantly, clinical evidence and animal experimental studies have shown that neutralizing antibodies, Western medicine, and traditional Chinese medicine slow down pulmonary fibrosis by regulating signaling pathways such as Wnt/β-catenin signaling, the immune balance of cell subsets such as Th1 and Th2, and receptors such as PD-1.

This review focuses on the role of CD4^+^T cell and CD8^+^T cell subsets in pulmonary inflammation and fibrosis, investigating their potential as therapeutic targets for managing pulmonary fibrosis. Future research on novel immunotherapy targets, the mechanisms influenced by the immune microenvironment, and the identification of potential biomarkers for immunotherapy will pave the way for broader and more effective clinical applications. Such advancements could enable precise regulation of various T lymphocyte subsets, slowing the progression of pulmonary fibrosis. This approach offers fresh directions, innovative ideas, and valuable insights for preventing and managing silicosis-related fibrosis.

TCR is the first signal for T cell activation. IFN-γ and IL-12 promote the expression of transcription factor T-bet by enhancing the STAT1 and STAT4 signals of T cells, driving the polarization of Th1 cells. IL-2 and IL-4 promote the expression of transcription factor GATA3 by enhancing the STAT5 signal of T cells, driving the polarization of Th2 cells. IL-2 and IL-9 promote the expression of transcription factor PU.1 by enhancing the STAT6 signal of T cells, driving the polarization of Th9 cells. IL-17 promotes the expression of transcription factor RORγT by enhancing the STAT3 signal of T cells, driving the polarization of Th17 cells. TGF-beta promotes the expression of transcription factor Foxp3 by enhancing the STAT5 signal of T cells, driving the polarization of Treg cells. ICOS promotes the expression of transcription factor Bcl-6 by enhancing the STAT3 signal of T cells, driving the polarization of Tfh cells. IL-2 drives the polarization of CD8^+^T cells by enhancing the STAT1 and STAT4 signals of CD8^+^T cells and promoting the expression of the transcription factor T-bet.

The binding of CD27 on T cells to CD70 on fibroblasts can reduce fibroblast polarization and collagen deposition alleviate the process of pulmonary fibrosis; TCR and CD28 on T cells bind to MHC and CD80 respectively, thereby slowing down the process of pulmonary fibrosis; Ivacaftor binds to CFTR and HU-308 binds to CB2 alleviate the process of pulmonary fibrosis; SP100030 slows down pulmonary fibrosis by inhibiting the NF-κB signaling pathway and DHZ slows down pulmonary fibrosis by inhibiting the Wnt/*β*-catenin signaling pathway. 4-1BB, PD-1, ICOS and OX40 on T cells bind to 4-1BB ligand, PD-L1/PD-L2, ICOSL and OX40L on antigen presenting cells promote the process of pulmonary fibrosis. Th1 cells inhibit pulmonary fibrosis by secreting IFN-γ, Th2 cells promote fibroblast polarization and pulmonary fibrosis by secreting IL-4 and IL-13, Th17 cells promote pulmonary fibrosis by secreting IL-17, and Treg cells promote pulmonary fibrosis in the early stage and inhibit it in the late stage.

## Glossary

APCs: Antigen presenting cells
BALF: Bronchoalveolar lavage fluid
Bcl-6: B-cell lymphoma 6 protein
CB2: Cannabinoid Receptor 2
CCR4+: Chemokine (C-C motif) Receptor 4
CCR7: C-C chemokine receptor type 7
CCR8: C-C chemokine receptor type 8
CD103: Integrin alpha E/CD103
CF: Cystic fibrosis
COPD: Chronic obstructive pulmonary disease
CTL: Cytotoxic T lymphocytes
CTLA-4: Cytotoxic T Lymphocyte-Associated Protein-4
CXCL10: C-X-C motif chemokine 10
DAPT: Dual Antigen-Presenting Therapy
DHZ: Dehydrozingerone
DLL: Delta-like ligand
ECM: ExtraCellular Matrix
EMT: Epithelial-mesenchymal transition
FN: Fibronectin
Foxp3: Forkhead box protein P3
GGD: Gan Cao Gan Jiang Tang
GZMH: Granzyme H
HYP: Hydroxyproline
ICOS: Inducible Synergistic Co-stimulation Molecules
IFN-γ: Interferon-gamma
Ig: Immunoglobulin
IL-12: Interleukin-12
IL-13: Interleukin-13
IL-17: Interleukin-17
IL-18: Interleukin-18
IL-1β: Interleukin-1β
IL-2: Interleukin-2
IL-21: Interleukin-21
IL-22: Interleukin-22
IL-4: Interleukin-4
IL-5: Interleukin-5
IL-6: Interleukin-6
IL-8: Interleukin-8
IL-9: Interleukin-9
IPF: Idiopathic pulmonary fibrosis
MCP-1: Monocyte chemotactic protein-1
MHC: Major histocompatibility complex
MMPs: Matrix metalloproteinases
NF-κB: Nuclear factor kappa-light-chain-enhancer of activated B cells
NKT: Natural killer T cells
PD- L2: Programmed Death Ligand 2
PD-1: Programmed Death-1
PD-L1: Programmed Death Ligand 1
PFD: Pirfenidone
PI3K: Phosphatidylinositol 3-kinase
RORγT: Retinoic acid receptor-related orphan receptor gamma t
SFRP2: Secreted frizzled-related protein 2
SiO_2_: Silicon Dioxide
SSc: Systemic sclerosis
STAT3: Signal transducer and activator of transcription 3
T-bet: T-box expressed in T cell
Tc1: Cytotoxic T cell 1
Tc2: Cytotoxic T cell 2
TCF: T cell factor
TCR: T cell receptor
T_EM_: Effective Memory T Cell
Tfh: T-Follicular helper cells
TGF-β: Transforming growth factor β
TGF-β3: Transforming growth factor β3
Th0: T helper type 0
Th1: T helper type 1
Th2: T helper type 2
Th9: T helper type 9
Th17: T helper type 17
TNF: Tumor necrosis factor
TRAF: TNF receptor-associated factor
Treg: Regulatory T cells
TRM: Tissue-resident memory T
TSLP: Thymic stromal lymphopoietin
WT: Wild-type
α-SMA: α-Smooth Muscle Actin
γδ T cells: Gamma delta T cell
